# Yeast proteinopathy models: a robust tool for deciphering the basis of neurodegeneration

**DOI:** 10.15698/mic2015.12.243

**Published:** 2015-11-26

**Authors:** Amit Shrestha, Lynn A. Megeney

**Affiliations:** 1 Ottawa Hospital Research Institute, Regenerative Medicine Program, Sprott Centre for Stem Cell Research, The Ottawa Hospital, Ottawa, Ontario, Canada.; 2 Department of Cellular and Molecular Medicine University of Ottawa, Ottawa, Ontario, Canada; 3 Department of Medicine, University of Ottawa, Ottawa, Ontario, Canada.

**Keywords:** yeast, neurodegeneration, proteinopathy, Hsp104, Yca1, TDP-43, α-synuclein

## Abstract

Protein quality control or proteostasis is an essential determinant of basic cell health and aging. Eukaryotic cells have evolved a number of proteostatic mechanisms to ensure that proteins retain functional conformation, or are rapidly degraded when proteins misfold or self-aggregate. Disruption of proteostasis is now widely recognized as a key feature of aging related illness, specifically neurodegenerative disease. For example, Alzheimer’s disease, Huntington’s disease, Parkinson’s disease and Amyotrophic Lateral Sclerosis (ALS) each target and afflict distinct neuronal cell subtypes, yet this diverse array of human pathologies share the defining feature of aberrant protein aggregation within the affected cell population. Here, we review the use of budding yeast as a robust proxy to study the intersection between proteostasis and neurodegenerative disease. The humanized yeast model has proven to be an amenable platform to identify both, conserved proteostatic mechanisms across eukaryotic phyla and novel disease specific molecular dysfunction. Moreover, we discuss the intriguing concept that yeast specific proteins may be utilized as bona fide therapeutic agents, to correct proteostasis errors across various forms of neurodegeneration.

## INTRODUCTION

The regulation of cellular protein levels is an indispensable feature that ensures fidelity of fundamental processes within all cell types. Although nascent peptide strands contain the requisite information to fold proteins into functional structures and confer biological activity, the dynamic nature of protein folding as well as the crowded cell environment pose significant barriers in maintaining protein homeostasis or proteostasis 1. Consequently, cells have evolved sophisticated protein quality control networks to ensure correct folding of nascent peptides, refolding of misfolded proteins or when required directed proteolysis via a number of conserved mechanisms 2.

Loss of proteostatic control has emerged as a common cellular pathology in seemingly disparate forms of neurodegenerative disease 3. Disruption to protein folding by various factors such as, mutations, errors in transcription/translation, environmental stress and age related decline can lead to the overwhelming of these quality control systems in susceptible neuron populations, resulting in the aggregation of misfolded proteins and their organization into larger aggregate structures 4. For example, various peptides (nascent and modified) can instinctively form detergent resistant cross-β amyloidogenic structures which are inherently associated with the pathology observed in various neurodegenerative disorders 5. These proteins include α-synuclein, the primary constituent in aggregate structures called Lewy bodies observed in Parkinson’s disease (PD) as well as other diseases termed synucleopathies. The more prevalent neurodegenerative disorder, Alzheimer’s disease (AD) is characterized by the presence of similar extracellular plaque structures containing accumulated β-amyloid peptide as well as intracellular buildup of a hyperphosphorylated microtubule associated protein referred to as tau. Similarly, intracellular cytotoxic aggregation of the huntingtin protein (Htt) is a hallmark feature of Huntington’s disease (HD) 5. More recently, highly ubiquitylated cytoplasmic inclusions of the TAR DNA-binding protein of 43 kDa (TDP-43) have been identified in neurons of Amyotrophic Lateral Sclerosis (ALS) affected patients 6. The amyloid structures formed by these various protein species have also been implicated in compromising the expression of proteostasis machinery. For instance, in mouse models of HD, increased polyQ aggregation resulted in a steady decline in the expression of the chaperones Hsp70 and the Hsp40 7.

A consensus has arisen supporting the concept that protein misfolding/aggregation and the progression of neurodegenerative disease are inherently linked. This model also suggests that ongoing aberrant protein misfolding and decline in proteostatic machinery function may accelerate the pathogenic cascade 3. The prevailing dogma argues that the proteostasis errors are a cell autonomous pathology, yet misfolded protein species of α-synuclein, β-amyloid and tau have been shown to be capable of cell to cell transmission similar to that of prions. These seed templates lead to the misfolding of the specific protein species, yet also initiate aggregate seeding of other proteins species, magnifying the proteostasis imbalance 8. Given these observations, there is an urgent unmet medical need to advance basic understanding of protein quality control systems and why perturbations in this core cellular activity propagates neurodegenerative disease.

In recent years, the field of neurodegeneration has derived significant benefit from the use of baker's yeast, *Saccharomyces cerevisiae,* to elucidate the molecular basis of various pathologies. 9,10,11. As the core proteostasis machinery is remarkably well conserved across eukaryotes, yeast has emerged as a tractable organism to model proteostasis alterations in neural disease 1,4,10,12. Here, we discuss the insights derived from the use of humanized yeast models and whether the yeast proteome itself may offer novel therapeutic avenues to treat and reverse otherwise implacable aggregation mediated disease.

## YEAST MODELS OF NEURODEGENERATION

The mislocalized accumulation of the pre-synaptic protein α-synuclein (α-syn) as a result of mutations or gene multiplications is a hallmark feature that defines PD and other disorders collectively termed synucleinopathies 13. α-syn was one of the first neurodegeneration related proteins to be characterized in the yeast model, which has greatly enhanced our understanding of the toxicity associated with PD. Indeed, the pathogenic prion-like spreading feature of α-syn oligomers and fibrils has generated considerable interest from both basic scientists who study aggregation control as well as those interested in disease pathogenesis 13,14. The very first study conducted by Outeiro and Lindquist 15 in yeast characterized α-syn toxicity as an outcome of its redistribution within the cell, which led to the formation of cytotoxic inclusions. This study demonstrated that toxicity was directly correlated with the level of α-syn expression, yet also established dysfunction in various cellular processes, namely, lipid droplet accumulation, impairment in the proteostasis machinery and defects in vesicle trafficking 15. Following this landmark report, numerous other studies utilizing yeast modeling of α-syn have added to the knowledge base, implicating defects in various cellular processes 10, as well as identifying normal cellular constituents that contribute to cytotoxicity, such as the correlation between mitochondria and reactive oxygen species formation that occurs in α-syn mediated cell death 16.

These studies highlight the great complexity in dealing with PD and neurodegenerative disorders in general, yet have confirmed the utility of the yeast system in addressing complex human disease pathology. Yeast models have also been instrumental in identifying other molecular determinants that modify α-syn cytotoxicity. In particular, the functional identification of the GTPase Rab1, as a suppressor of α-syn toxicity, stemmed from initial studies in yeast 17. The observation that α-syn accumulation leads to ER-Golgi trafficking defects, which is present in PD and many other neurodegenerative disorders, led to the subsequent utilization of yeast overexpression libraries to screen for modifiers of α-syn toxicity. The yeast protein Ypt1; a Rab family related GTPase, was observed to directly interact with α-syn inclusions and suppress α-syn toxicity. The protective function of Ypt1 was also observed to be phylogenetically conserved, as its mammalian homolog Rab1 was able to rescue the loss of dopaminergic neuron loss in PD models in both *Drosophila *and *C. elegans *17. Subsequently, other members of the Rab family of GTPases have been also shown to suppress α-syn toxicity. For example, the highly expressed presynaptic protein RAB3A and the post-Golgi vesicle associated protein, RAB8A have been demonstrated to ameliorate vesicle trafficking defects associated with α-syn expression. The heavy reliance of dopaminergic neurons on vesicular trafficking is also suggested to be the reason that this subset of neurons is affected the most in PD 18. More recently, α-syn expression has been shown to imbalance Rab homeostasis that results in Golgi fragmentation observed in PD. Golgi fragmentation was demonstrated to correlate with the expression levels of Rab 1, 2 and 8. Overexpression of Rab 1 and 8 and the ablation of Rab2 expression rescued the fragmentation phenotype 19.

In addition to Rab GTPases, studies using similar approaches continue to identify novel protein mediators of α-syn related cytotoxicity in PD, including endonuclease G (endoG) and the sorting protein VPS35 20. The cytotoxic mislocalization of the mitochondrial nuclease endoG in the nucleus of dopaminergic neurons in PD patients inspired its characterization in yeast PD models. In yeast, it was observed that expression of α-syn leads to DNA damage, which is mediated by endoG. This observation was further verified in fly models which showed that suppressing endoG increased the survival of α-syn expressing flies, implicating endoG as a critical mediator of α-syn toxicity and a potential target for therapeutic development 20. VPS35 has also been shown to ameliorate neurodegeneration and antagonize α-syn inclusion formation. This is of particularly interest as mutations in VPS35 are linked to PD 21. Increased gene expression of the translation initiation factor EIF4G1 has also been linked to protein misfolding as well as cases of familial PD 22. Overexpression of the yeast homolog of EIF4G1, TIF4631, was toxic to cells lacking the *vps35* gene suggesting that VPS35 is protective against toxicity relating to EIF4G1 upregulation. Likewise, the PD-associated D620N mutation in VPS35 also had a similar effect. This genetic interaction between VPS35 and EIF4G1 was further confirmed in neurons of both *C. elegans* and transgenic mouse models 23. Furthermore, loss of either VPS35 or TIF4631 expression in yeast reduces the survival rate against α-syn toxicity. Finally, staining of NeuN in the hippocampus suggested that VPS35 upregulation is protective against α-syn associated neurodegeneration 23. As such, these studies highlight the ease and accuracy of the yeast model system to define the molecular pathogenesis of human PD.

Understanding the cellular etiology of ALS, a fatal and intractable neurodegenerative disorder, has also benefitted greatly by use of yeast model systems 9,10. A superb example of one such ALS related protein is TDP-43 (TAR DNA binding protein 43). TDP-43 was originally described as a regulator of RNA metabolism in the nucleus and has also been shown to have a cytoplasmic role in anterograde transport of trafficking mRNAs in neurons. Nevertheless, TDP-43 has been found to be a common cytoplasmic inclusion protein in ALS affected individuals 9,24. In addition, TDP-43 inclusions have been linked to many other neurodegenerative disorders such as Inclusion Body Myopathy with Paget disease of the bone and frontotemporal dementia (IBMPFD) as well as AD, PD and HD 25.

The first yeast TDP-43 proteinopathy model by Johnson *et. al.* 26 characterized TDP-43 to be nucleus specific and that overexpression of the protein led to its mislocalization into the cytoplasm where it formed aggregates, reducing overall cell survival. Interestingly, TDP-43 inclusions were observed to differ from those of the expanded polyglutamine Htt protein associated with HD, as TDP-43 aggregates were able to solubilize and were not resistant to detergent denaturation. This study used a deletion mutant approach to dissect the structural requirements within the protein to identify region(s) that were responsible for aggregation propensity and cytotoxicity and deduced that the C terminus of the protein was responsible for driving aggregation 26. Further characterization of TDP-43 in yeast identified TDP-43 as having the innate ability to aggregate as pure TDP-43 readily formed inclusions, which were structurally identical to aggregates in degenerating neurons of patients with ALS and frontotemporal lobar degeneration with ubiquitin- and TDP-43-positive inclusions (FTLD-U). Use of the yeast model revealed that several reported pathogenic mutations linked to ALS mapped to the C-terminus of TDP-43, which accelerated TDP-43 aggregation and decreased survival 27. These initial discoveries of TDP-43 disease biology have now been validated in various experimental models 9,28.

The success of modifier gene discovery in α-syn yeast models of PD has also propelled the use of similar genome wide screens to elucidate TDP-43 induced toxicity, i.e. yeast overexpression screens have led to the identification of a pool of 40 genes that modify TDP-43 toxicity. This pool of factors contained the Poly (A) binding protein (Pab1) - binding protein, Pbp1 as a specific enhancer of TDP-43 toxicity; overexpression of Pbp1 increased TDP-43 toxicity whereas as Δ*pbp1* cells showed increased cell survival upon TDP-43 overexpression 29. Interestingly, Pbp1 is an ortholog of the human ataxin-2 gene (*atxn2*), which has been implicated in spinocerebellar ataxia type 2 (SCA2) that is caused by glutamine expansion within the polyQ tract. This interaction between TDP-43 and Pbp1/Atx2 was further validated in *Drosophila* and humans. Moreover, the physical interaction between TDP-43 and Atx2 was shown to be dependent on RNA binding, as mutations within the RRMs as well as RNase treatment, dissolve the interaction between Atx2 and TDP-43 29. Atx2, which is normally granular or diffused within the cytoplasm, was observed to be aggregated in the spinal cord neurons of ALS patients, prompting the speculation that the TDP-43-Atx2 may have a causative link in ALS. Consequently, analysis of polyglutamine length of Atx2 in ALS patients led to the conclusion that the presence of an intermediate length glutamine expansion (27-33Q) was associated with an increased risk of ALS, a conclusion that was matched by the early age of onset in affected individuals 29.

Building on this early work, recent studies have implicated the Pab1 protein itself (an Atx2 interactor and component of cytoplasmic stress granules where mislocalized TDP-43 accumulates), as having a role in TDP-43 mediated toxicity. Using the fly model, investigators have shown that an Atx2 mutant lacking the PAM1 motif (through which it binds Pab1), cannot interact with TDP-43 and confer toxicity in the retina. Similarly, human PABPC1 was observed to be mislocalized in ALS patients 30. This study also led to the identification of the yeast ORF *YGR054W,* whose human homolog is EIF2A, as having physical and genetic interactions with multiple TDP-43 modifying genes. Further investigation correlated TDP-43 expression with the level of EIF2A phosphorylation and that blocking EIF2A phosphorylation by knocking down the PERK homolog PEK, was able to rescue TDP-43 toxicity in *Drosophila*. Collectively, these studies raise the tantalizing premise that TDP-43 toxicity may be moderated, by modulating the base level of EIF2A phosphorylation 30.

TDP-43 toxicity appears to also segregate with modifications in DNA structure. Here, Armakola *et. al.* 31 used yeast deletion screens to identify the RNA lariat debranching enzyme, Dbr1, as a conditioner of TDP-43 toxicity. Dbr1 is a phylogenetically conserved phosphodiesterase that is required for RNA degradation; and *dbr1* deletion mitigated the toxic effects of wildtype TDP-43 and the mutant TDP-43 Q331K expression. This suppression of TDP-43 toxicity was also validated in the human M17 neuroblastoma cell line and primary neurons using a siRNA-based knockdown approach, suggesting that inhibiting the enzymatic activity of Dbr1 is sufficient to reduce TDP-43 toxicity 31. Using a Dbr1 mutant panel, the authors reported that limiting Dbr1 enzymatic activity led to RNA lariat accumulation, directly correlated with TDP-43 toxicity. Fluorescence imaging in yeast showed that TDP-43 foci co-localized with intronic lariats of the *Act1* gene in the cytoplasm and the suppression of TDP-43 toxicity in Δ*dbr1 *results from interactions of TDP-43 with the accumulated intronic lariats. The postulate derived from these experiments implied that TDP-43 associated toxicity in ALS and FTLD-U arose from a loss of essential TDP-43 RNA interactions 31,32. The identification of TDP-43 as a RNA binding protein inspired efforts to test other RNA binding proteins as ALS relevant targets, among which FUS/TLS, TAF15, EWSR1, HNRNPA1 and HNRNPA2B1 have received considerable attention. Functional analyses of these proteins in yeast and other models have shown that, together with TDP-43, these proteins constitute an assemblage of factors with innate aggregation propensity, a characteristic which is accelerated in disease-linked forms 32,33,34. Furthermore, studies using a similar deletion approach recently identified that mutations within the microtubule associated protein, profilin 1 (PFN1), which disrupts its novel function in stress granule dynamics can ablate its ability to mediate ALS pathology 35. Such studies suggest the yeast model remains a focal point to dissect the functional role of these candidate genes as well as identify additional candidates in ALS and other related pathologies 9.

The prior discussion has focused on the role of yeast models as a test bed to explore disease mechanisms in α-syn linked PD and TDP-43 linked ALS, yet yeast has served as an ideal platform to study the cell biology of various aggregation prone proteins that characterize neurodegeneration. Of note, is the recent elucidation that manipulation of the ubiquitin protease system (UPS) in yeast (by enhancing the expression level of Cdc48 and Vms1) can curtail UBB^+1^ induced mitochondrial stress, which is a hallmark feature of AD pathology 36, This study exemplifies the matchless speed and ease of application afforded by use of the yeast model to address outstanding issues in neurodegenerative disease 10.

## YEAST PROTEIN DELIVERY AS A MEANS TO COMBAT NEURODEGENERATION 

Much effort has focused on defining genetic susceptibility in neurodegenerative disease, yet this line of investigation has failed to provide new or effective clinical interventions. Indeed, the capacity to actively reverse aggregation and control protein misfolding will be an essential attribute when considering therapeutic solutions for proteinopathies. Provocative evidence now suggests that yeast specific proteins have the capacity to degrade aggregate prone human disease proteins. Although speculative at this point in time, the data does support the conjecture that yeast proteins may be eventually employed to combat human neurodegeneration. In this regard, the yeast specific Hsp104 chaperone disaggregase has shown considerable promise 37.

Hsp104 is an AAA+ chaperone of the heat shock protein family which is known for its ability to completely remodel denatured aggregates, conferring “thermotolerance” in yeast. Structurally, Hsp104 is hexameric pore-like structure consisting of an N-terminal domain, a middle domain (MD) flanked by two AAA (AAA-1/2) domains and a C-terminal domain. ATP hydrolysis at the two AAA domains is required for substrate translocation through the pore. The unique M-domain, which is absent in other AAA+ proteins, is suggested to be the molecular switch which mediates the ATPase activity of Hsp104 and co-ordinates interaction with other chaperones of the Hsp70 and Hsp40 families 38,39. Dissolution of misfolded peptides by Hsp104 is dictated by ATP hydrolysis at the two AAA domains, resulting in a “peristaltic” translocation of the substrate through the pore leading to the release of the native peptide for proper refolding at the C terminus by other chaperones. Hsp104 is also essential for the propagation of prions, which are structurally amyloid in nature and considered to be beneficial for yeast to adapt and survive in varying environments 39.

**Figure 1:**
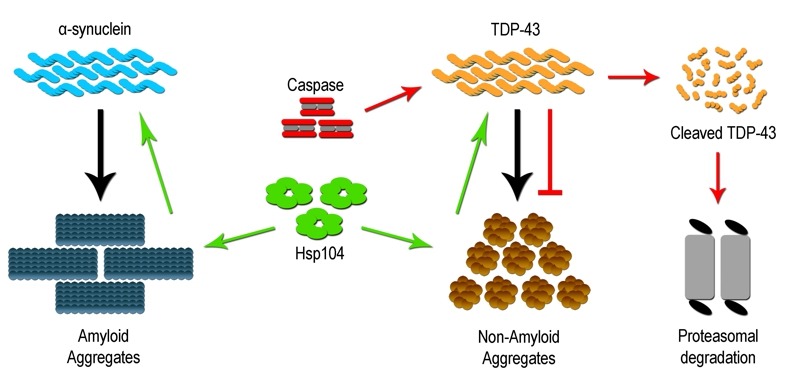
FIGURE 1: Therapeutic strategies to limit protein aggregation based disorders. Many neurodegeneration disorders are characterized by increased protein aggregation of diseased associated proteins like α-synuclein and TDP-43. The yeast specific disaggregase Hsp104 and its variants (green) have been shown to be an effective in limiting inclusion formation and accumulation. Hsp104 acts on the inclusion to dissolve and restore them to the properly folded soluble form (TDP-43) as well as eliminate toxic soluble oligomeric forms (α-synuclein) of the protein. Additionally, caspase/metacaspase family of proteases (red) have also been shown to limit inclusion formation by directly cleaving the disease associated protein (TDP-43), which is subsequently degraded by the proteasome.

Metazoans lack the presence of a clear Hsp104 ortholog, yet despite its absence Hsp104 can be stably expressed and can rescue protein aggregation and prevent neurodegeneration 30. For example, Hsp104 overexpression within a rat model for PD was able to prevent dopaminergic neuron loss. This rescue was attributed to the ability of Hsp104 to disassemble toxic oligomers of α-syn *in vitro* as well as prevent α-syn inclusion formation *in vivo* 40. Hsp104 has been shown to have a wide clientele of disease associated substrates, including both wildtype and mutant protein forms, which can be dissembled and resolubilized within the cell. However, dissolution of prion substrates, such as Sup35 in yeast, was more challenging for Hsp104 than other misfolded substrates, suggesting a need for a better understanding of the Hsp104 mechanism and activity 37,41. Subsequent studies have focused on defining variants of Hsp104 that may impart enhanced disaggregase activity, and efforts to date have identified variants that modify proteinopathy associated with gain of function mutations for α-syn, TDP-43 (Figure 1) and other TDP-43 related RNA binding proteins such as FUS. Further examination *in vitro *revealed that the enhanced disaggregase activity was dictated by amino acid identity within the middle domain of Hsp104, and key mutations in this region increased chaperone collaboration leading to disaggregation of a wide range of substrates 37,42,43. Therefore, deployment of Hsp104 variants may offer a novel means to mitigate aggregation based disorders. What remains more speculative is whether Hsp104 can be engineered to attack very precise or structurally unique aggregates 37.

The unexpected role of Hsp104 in mitigating human aggregate-sensitive disease proteins raises the prospect that additional unforeseen regulatory mechanisms may exist to manage this critical aspect of cell biology. As such, a novel proteostasis role has been identified for the clan C/D family of proteases, which include metacaspases and caspases. While this protease clan is typically associated with induction of programmed cell death, through broad range protein destruction, a growing body of evidence suggests that discrete activation of these proteases is essential for numerous complex cell behaviors, independent of inducing cell death 44,45. Evidence supporting this claim originates from the observation that the loss of yeast metacaspase, Yca1 (also known as Mca1) results in an increased retention of insoluble aggregated material and decreased cell fitness 46,47. This provocative study also confirmed Yca1 formed stable interactions with known chaperone proteins such as Hsp40/70 and Cdc48/VCP 46. Subsequent studies have affirmed the involvement of Yca1 in protein quality control, demonstrating that Yca1 regulates the composition of the insoluble proteome, by moderating the level of proteins that manage translation and ribosome biogenesis during stress 48.

Most recently, Hill *et al.* 49 identified Yca1 as a critical component in the control of age related protein aggregation. Using synthetic genetic array (SGA) methodology, these investigators conducted an unbiased genome wide screen to identify regulatory factors that limit dispersal of protein aggregates during replicative cell aging, i.e. retention of aggregates in the mother cell during division. This study established that Yca1 shuttled between quality control compartments and limited protein aggregation in the daughter cell 49. Given the strong association of Yca1 with the chaperone machinery, combinatorial delivery of this metacaspase along with Hsp104 may offer a unique mechanism to restrain aggregate toxicity in affected human neurons.

Admittedly, the delivery of yeast proteins as a therapeutic intervention to treat human neurodegeneration is an entirely speculative proposition. However, the emerging data for Hsp104 indicates that measured delivery of this protein in preclinical animal models of PD and Huntington’s disease has no observable off target toxicity 40,50,51. Similar concerns should be noted for metacaspase use as this protease has yet to be expressed and tested in any mammalian system.

The discovery in yeast, that a metacaspase subdues toxic aggregate formation, raises an interesting prospect, i.e. that mammalian/human caspase proteins may have retained similar beneficial proteostatic function(s). Early studies characterizing cellular pathology in neurodegenerative disease have noted that affected and dying neurons display robust elevation in effector caspase activity (caspase 3 and 6), with subcellular distribution to protein inclusions 52,53. The prevailing assumption is that this caspase activity profile is causative in the generation of disease linked inclusions, in keeping with the known pro-apoptotic role for caspase enzymes. Not surprisingly, these observations have led to the hypothesis that targeted inhibition of caspase activity may provide a viable therapeutic intervention to limit cell death and dysfunction across a broad spectrum of aggregate associated neural disease 54. Despite the widespread appeal that caspase activity acts as a harbinger of neural degeneration, the concept defies a wealth of opposing data, demonstrating that caspase function is vital for neurogenesis/neural cell differentiation, synaptic remodeling and higher brain function(s) 55,56. Indeed, preliminary investigations have reported that targeted cleavage of TDP-43 by caspase 3 results in reduced TDP-43 aggregation [Figure 1]. This study also demonstrated that cleavage-resistant forms of TDP-43 were more toxic than the resulting cleavage products, suggesting that caspase mediated degradation is critical for reducing TDP-43 toxicity and perhaps limiting the advent of ALS 57. As such, the elevation of effector caspase activation in diseased cells may simply reflect an adaptive response whereby caspases engage to limit deposition or expansion of toxic aggregates. If one makes the reasonable assumption that caspase enzymes have retained the yeast metacaspase function through evolution, then a probable model would predict that caspases similarly integrate with chaperones and folding/refolding machinery to restrain aggregate deposition and growth.

## CONCLUSION

In conclusion, we have demonstrated that deployment of the yeast model has provided exceptional advances in the study of neurodegenerative disorders including PD and ALS. Similar concerted efforts using yeast proteinopathy models have advanced understanding of disease related proteins such as tau, Aβ and prions. Likewise, the concept that yeast specific proteins, such as Hsp104 and/or Yca1, may be engineered to combat toxic human aggregates opens the door to entirely novel avenues of therapeutic intervention. The use of yeast proteins as therapeutic agents needs to be approached with considerable caution, yet the early studies in relevant animal models support the basic premise. Clearly, the ease and flexibility of the yeast model will ensure continued use of this eukaryotic cell as a preferred means to interrogate and define the molecular pathology of neurodegeneration.

## References

[1] Hartl FU, Bracher A, Hayer-Hartl M (2011). Molecular chaperones in protein folding and proteostasis.. Nature.

[2] Winkler J, Tyedmers J, Bukau B, Mogk A (2012). Chaperone networks in protein disaggregation and prion propagation.. Journal of structural biology.

[3] Morimoto RI (2008). Proteotoxic stress and inducible chaperone networks in neurodegenerative disease and aging.. Genes & development.

[4] Tyedmers J, Mogk A, Bukau B (2010). Cellular strategies for controlling protein aggregation.. Nature reviews Molecular cell biology.

[5] Ross CA, Poirier MA (2004). Protein aggregation and neurodegenerative disease.. Nature medicine.

[6] Neumann M, Sampathu DM, Kwong LK, Truax AC, Micsenyi MC, Chou TT, Bruce J, Schuck T, Grossman M, Clark CM, McCluskey LF, Miller BL, Masliah E, Mackenzie IR, Feldman H, Feiden W, Kretzschmar HA, Trojanowski JQ, Lee VM (2006). Ubiquitinated TDP-43 in frontotemporal lobar degeneration and amyotrophic lateral sclerosis.. Science.

[7] Hay DG, Sathasivam K, Tobaben S, Stahl B, Marber M, Mestril R, Mahal A, Smith DL, Woodman B, Bates GP (2004). Progressive decrease in chaperone protein levels in a mouse model of Huntington's disease and induction of stress proteins as a therapeutic approach.. Human molecular genetics.

[8] Polymenidou M, Cleveland DW (2012). Prion-like spread of protein aggregates in neurodegeneration.. The Journal of experimental medicine.

[9] Figley MD, Gitler AD (2013). Yeast genetic screen reveals novel therapeutic strategy for ALS.. Rare diseases.

[10] Tenreiro S, Munder MC, Alberti S, Outeiro TF (2013). Harnessing the power of yeast to unravel the molecular basis of neurodegeneration.. Journal of neurochemistry.

[11] Braun RJ, Buttner S, Ring J, Kroemer G, Madeo F (2010). Nervous yeast: modeling neurotoxic cell death.. Trends in biochemical sciences.

[12] Braun RJ (2015). Ubiquitin-dependent proteolysis in yeast cells expressing neurotoxic proteins.. Frontiers in molecular neuroscience.

[13] Lashuel HA, Overk CR, Oueslati A, Masliah E (2013). The many faces of alpha-synuclein: from structure and toxicity to therapeutic target.. Nature reviews Neuroscience.

[14] Alberti S, Halfmann R, King O, Kapila A, Lindquist S (2009). A systematic survey identifies prions and illuminates sequence features of prionogenic proteins.. Cell.

[15] Outeiro TF, Lindquist S (2003). Yeast cells provide insight into alpha-synuclein biology and pathobiology.. Science.

[16] Buttner S, Bitto A, Ring J, Augsten M, Zabrocki P, Eisenberg T, Jungwirth H, Hutter S, Carmona-Gutierrez D, Kroemer G, Winderickx J, Madeo F (2008). Functional mitochondria are required for alpha-synuclein toxicity in aging yeast.. The Journal of biological chemistry.

[17] Cooper AA, Gitler AD, Cashikar A, Haynes CM, Hill KJ, Bhullar B, Liu K, Xu K, Strathearn KE, Liu F, Cao S, Caldwell KA, Caldwell GA, Marsischky G, Kolodner RD, Labaer J, Rochet JC, Bonini NM, Lindquist S (2006). Alpha-synuclein blocks ER-Golgi traffic and Rab1 rescues neuron loss in Parkinson's models.. Science.

[18] Gitler AD, Bevis BJ, Shorter J, Strathearn KE, Hamamichi S, Su LJ, Caldwell KA, Caldwell GA, Rochet JC, McCaffery JM, Barlowe C, Lindquist S (2008). The Parkinson's disease protein alpha-synuclein disrupts cellular Rab homeostasis.. Proceedings of the National Academy of Sciences of the United States of America.

[19] Rendon WO, Martinez-Alonso E, Tomas M, Martinez-Martinez N, Martinez-Menarguez JA (2013). Golgi fragmentation is Rab and SNARE dependent in cellular models of Parkinson's disease.. Histochemistry and cell biology.

[20] Buttner S, Habernig L, Broeskamp F, Ruli D, Vogtle FN, Vlachos M, Macchi F, Kuttner V, Carmona-Gutierrez D, Eisenberg T, Ring J, Markaki M, Taskin AA, Benke S, Ruckenstuhl C, Braun R, Van den Haute C, Bammens T, van der Perren A, Frohlich KU, Winderickx J, Kroemer G, Baekelandt V, Tavernarakis N, Kovacs GG, Dengjel J, Meisinger C, Sigrist SJ, Madeo F (2013). Endonuclease G mediates alpha-synuclein cytotoxicity during Parkinson's disease.. The EMBO journal.

[21] Vilarino-Guell C, Wider C, Ross OA, Dachsel JC, Kachergus JM, Lincoln SJ, Soto-Ortolaza AI, Cobb SA, Wilhoite GJ, Bacon JA, Behrouz B, Melrose HL, Hentati E, Puschmann A, Evans DM, Conibear E, Wasserman WW, Aasly JO, Burkhard PR, Djaldetti R, Ghika J, Hentati F, Krygowska-Wajs A, Lynch T, Melamed E, Rajput A, Rajput AH, Solida A, Wu RM, Uitti RJ (2011). VPS35 mutations in Parkinson disease.. American journal of human genetics.

[22] Chartier-Harlin MC, Dachsel JC, Vilarino-Guell C, Lincoln SJ, Lepretre F, Hulihan MM, Kachergus J, Milnerwood AJ, Tapia L, Song MS, Le Rhun E, Mutez E, Larvor L, Duflot A, Vanbesien-Mailliot C, Kreisler A, Ross OA, Nishioka K, Soto-Ortolaza AI, Cobb SA, Melrose HL, Behrouz B, Keeling BH, Bacon JA, Hentati E, Williams L, Yanagiya A, Sonenberg N, Lockhart PJ, Zubair AC (2011). Translation initiator EIF4G1 mutations in familial Parkinson disease.. American journal of human genetics.

[23] Dhungel N, Eleuteri S, Li LB, Kramer NJ, Chartron JW, Spencer B, Kosberg K, Fields JA, Stafa K, Adame A, Lashuel H, Frydman J, Shen K, Masliah E, Gitler AD (2015). Parkinson's disease genes VPS35 and EIF4G1 interact genetically and converge on alpha-synuclein.. Neuron.

[24] Alami NH, Smith RB, Carrasco MA, Williams LA, Winborn CS, Han SS, Kiskinis E, Winborn B, Freibaum BD, Kanagaraj A, Clare AJ, Badders NM, Bilican B, Chaum E, Chandran S, Shaw CE, Eggan KC, Maniatis T, Taylor JP (2014). Axonal transport of TDP-43 mRNA granules is impaired by ALS-causing mutations.. Neuron.

[25] Geser F, Martinez-Lage M, Kwong LK, Lee VM, Trojanowski JQ (2009). Amyotrophic lateral sclerosis, frontotemporal dementia and beyond: the TDP-43 diseases.. Journal of neurology.

[26] Johnson BS, McCaffery JM, Lindquist S, Gitler AD (2008). A yeast TDP-43 proteinopathy model: Exploring the molecular determinants of TDP-43 aggregation and cellular toxicity.. Proceedings of the National Academy of Sciences of the United States of America.

[27] Johnson BS, Snead D, Lee JJ, McCaffery JM, Shorter J, Gitler AD (2009). TDP-43 is intrinsically aggregation-prone, and amyotrophic lateral sclerosis-linked mutations accelerate aggregation and increase toxicity.. The Journal of biological chemistry.

[28] Braun RJ, Sommer C, Carmona-Gutierrez D, Khoury CM, Ring J, Buttner S, Madeo F (2011). Neurotoxic 43-kDa TAR DNA-binding protein (TDP-43) triggers mitochondrion-dependent programmed cell death in yeast.. The Journal of biological chemistry.

[29] Elden AC, Kim HJ, Hart MP, Chen-Plotkin AS, Johnson BS, Fang X, Armakola M, Geser F, Greene R, Lu MM, Padmanabhan A, Clay-Falcone D, McCluskey L, Elman L, Juhr D, Gruber PJ, Rub U, Auburger G, Trojanowski JQ, Lee VM, Van Deerlin VM, Bonini NM, Gitler AD (2010). Ataxin-2 intermediate-length polyglutamine expansions are associated with increased risk for ALS.. Nature.

[30] Kim HJ, Raphael AR, LaDow ES, McGurk L, Weber RA, Trojanowski JQ, Lee VM, Finkbeiner S, Gitler AD, Bonini NM (2014). Therapeutic modulation of eIF2alpha phosphorylation rescues TDP-43 toxicity in amyotrophic lateral sclerosis disease models.. Nature genetics.

[31] Armakola M, Higgins MJ, Figley MD, Barmada SJ, Scarborough EA, Diaz Z, Fang X, Shorter J, Krogan NJ, Finkbeiner S, Farese Jr RV, Gitler AD (2012). Inhibition of RNA lariat debranching enzyme suppresses TDP-43 toxicity in ALS disease models.. Nature genetics.

[32] King OD, Gitler AD, Shorter J (2012). The tip of the iceberg: RNA-binding proteins with prion-like domains in neurodegenerative disease.. Brain research.

[33] Couthouis J, Hart MP, Shorter J, DeJesus-Hernandez M, Erion R, Oristano R, Liu AX, Ramos D, Jethava N, Hosangadi D, Epstein J, Chiang A, Diaz Z, Nakaya T, Ibrahim F, Kim HJ, Solski JA, Williams KL, Mojsilovic-Petrovic J, Ingre C, Boylan K, Graff-Radford NR, Dickson DW, Clay-Falcone D, Elman L, McCluskey L, Greene R, Kalb RG, Lee VM, Trojanowski JQ (2011). A yeast functional screen predicts new candidate ALS disease genes.. Proceedings of the National Academy of Sciences of the United States of America.

[34] Kim HJ, Kim NC, Wang YD, Scarborough EA, Moore J, Diaz Z, MacLea KS, Freibaum B, Li S, Molliex A, Kanagaraj AP, Carter R, Boylan KB, Wojtas AM, Rademakers R, Pinkus JL, Greenberg SA, Trojanowski JQ, Traynor BJ, Smith BN, Topp S, Gkazi AS, Miller J, Shaw CE, Kottlors M, Kirschner J, Pestronk A, Li YR, Ford AF, Gitler AD (2013). Mutations in prion-like domains in hnRNPA2B1 and hnRNPA1 cause multisystem proteinopathy and ALS.. Nature.

[35] Figley MD, Bieri G, Kolaitis RM, Taylor JP, Gitler AD (2014). Profilin 1 associates with stress granules and ALS-linked mutations alter stress granule dynamics.. The Journal of neuroscience : the official journal of the Society for Neuroscience.

[36] Braun RJ, Sommer C, Leibiger C, Gentier RJ, Dumit VI, Paduch K, Eisenberg T, Habernig L, Trausinger G, Magnes C, Pieber T, Sinner F, Dengjel J, van Leeuwen FW, Kroemer G, Madeo F (2015). Accumulation of Basic Amino Acids at Mitochondria Dictates the Cytotoxicity of Aberrant Ubiquitin.. Cell reports.

[37] Jackrel ME, Shorter J (2015). Engineering enhanced protein disaggregases for neurodegenerative disease.. Prion.

[38] Desantis ME, Shorter J (2012). The elusive middle domain of Hsp104 and ClpB: location and function.. Biochimica et biophysica acta.

[39] Vashist S, Cushman M, Shorter J (2010). Applying Hsp104 to protein-misfolding disorders.. Biochemistry and cell biology = Biochimie et biologie cellulaire.

[40] Lo Bianco C, Shorter J, Regulier E, Lashuel H, Iwatsubo T, Lindquist S, Aebischer P (2008). Hsp104 antagonizes alpha-synuclein aggregation and reduces dopaminergic degeneration in a rat model of Parkinson disease.. The Journal of clinical investigation.

[41] Duennwald ML, Echeverria A, Shorter J (2012). Small heat shock proteins potentiate amyloid dissolution by protein disaggregases from yeast and humans.. PLoS biology.

[42] Jackrel ME, DeSantis ME, Martinez BA, Castellano LM, Stewart RM, Caldwell KA, Caldwell GA, Shorter J (2014). Potentiated Hsp104 variants antagonize diverse proteotoxic misfolding events.. Cell.

[43] Jackrel ME, Shorter J (2014). Potentiated Hsp104 variants suppress toxicity of diverse neurodegenerative disease-linked proteins.. Disease models & mechanisms.

[44] Dick SA, Megeney LA (2013). Cell death proteins: an evolutionary role in cellular adaptation before the advent of apoptosis.. BioEssays : news and reviews in molecular, cellular and developmental biology.

[45] Kuranaga E (2012). Beyond apoptosis: caspase regulatory mechanisms and functions in vivo.. Genes to cells : devoted to molecular & cellular mechanisms.

[46] Lee RE, Brunette S, Puente LG, Megeney LA (2010). Metacaspase Yca1 is required for clearance of insoluble protein aggregates.. Proceedings of the National Academy of Sciences of the United States of America.

[47] Lee RE, Puente LG, Kaern M, Megeney LA (2008). A non-death role of the yeast metacaspase: Yca1p alters cell cycle dynamics.. PloS one.

[48] Shrestha A, Puente LG, Brunette S, Megeney LA (2013). The role of Yca1 in proteostasis. Yca1 regulates the composition of the insoluble proteome.. Journal of proteomics.

[49] Hill SM, Hao X, Liu B, Nystrom T (2014). Life-span extension by a metacaspase in the yeast Saccharomyces cerevisiae.. Science.

[50] Vacher C, Garcia-Oroz L, Rubinsztein DC (2005). Overexpression of yeast hsp104 reduces polyglutamine aggregation and prolongs survival of a transgenic mouse model of Huntington's disease.. Human molecular genetics.

[51] Perrin V, Regulier E, Abbas-Terki T, Hassig R, Brouillet E, Aebischer P, Luthi-Carter R, Deglon N (2007). Neuroprotection by Hsp104 and Hsp27 in lentiviral-based rat models of Huntington's disease.. Molecular therapy : the journal of the American Society of Gene Therapy.

[52] Shimohama S, Tanino H, Fujimoto S (1999). Changes in caspase expression in Alzheimer's disease: comparison with development and aging.. Biochemical and biophysical research communications.

[53] LeBlanc A, Liu H, Goodyer C, Bergeron C, Hammond J (1999). Caspase-6 role in apoptosis of human neurons, amyloidogenesis, and Alzheimer's disease.. The Journal of biological chemistry.

[54] LeBlanc AC (2013). Caspase-6 as a novel early target in the treatment of Alzheimer's disease.. The European journal of neuroscience.

[55] D'Amelio M, Sheng M, Cecconi F (2012). Caspase-3 in the central nervous system: beyond apoptosis.. Trends in neurosciences.

[56] McLaughlin B, Hartnett KA, Erhardt JA, Legos JJ, White RF, Barone FC, Aizenman E (2003). Caspase 3 activation is essential for neuroprotection in preconditioning.. Proceedings of the National Academy of Sciences of the United States of America.

[57] Suzuki H, Lee K, Matsuoka M (2011). TDP-43-induced death is associated with altered regulation of BIM and Bcl-xL and attenuated by caspase-mediated TDP-43 cleavage.. The Journal of biological chemistry.

